# Supplementary material for: A multiplexable TALE-based binary expression system for in vivo cellular interaction studies
Source: Nat Commun. 2017 Nov 21;8:1663. doi: 10.1038/s41467-017-01592-3 (PMC5698491; doi:10.1038/s41467-017-01592-3)
Supplement: Supplementary file 1 — Supplementary Information [file 41467_2017_1592_MOESM1_ESM.pdf]

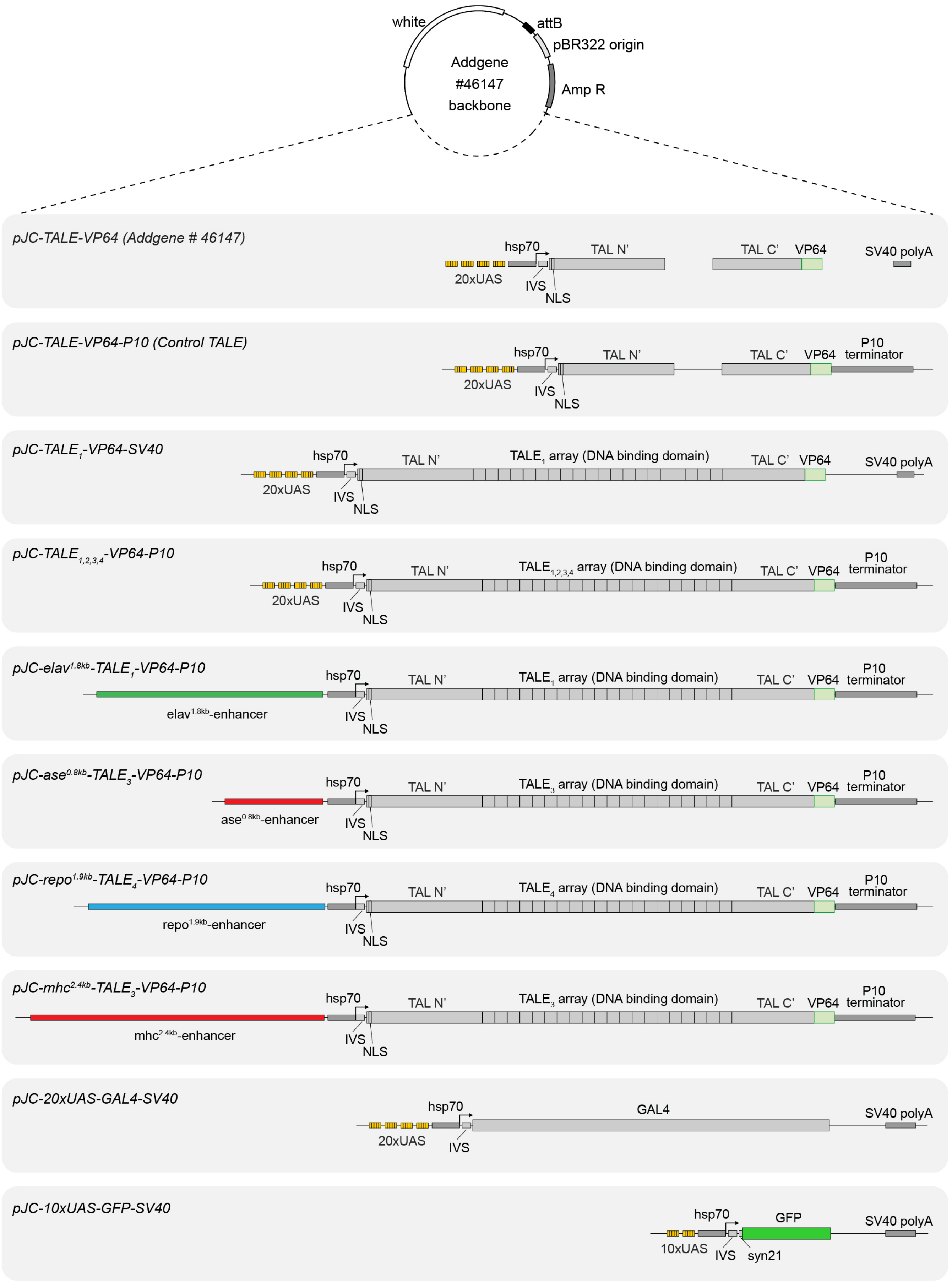

Supplementary Figure 1. Constructs based on the pJC-TALE-VP64-SV40 plasmid backbone. The JC-TALE-VP64-SV40 plasmid (Addgene vector #46147) was used to generate all TALE drivers as well as the 20 x UAS-GAL4 and the 10 x UAS-GFP constructs. Modifications to the original vector included replacing the SV40 terminator with a P10 terminator, introducing the custom TALE arrays, replacing the 20 x UAS enhancer with *Drosophila* tissue specific enhancers, and replacing the entire TALE cassette with GAL4 or GFP (see Online Methods).

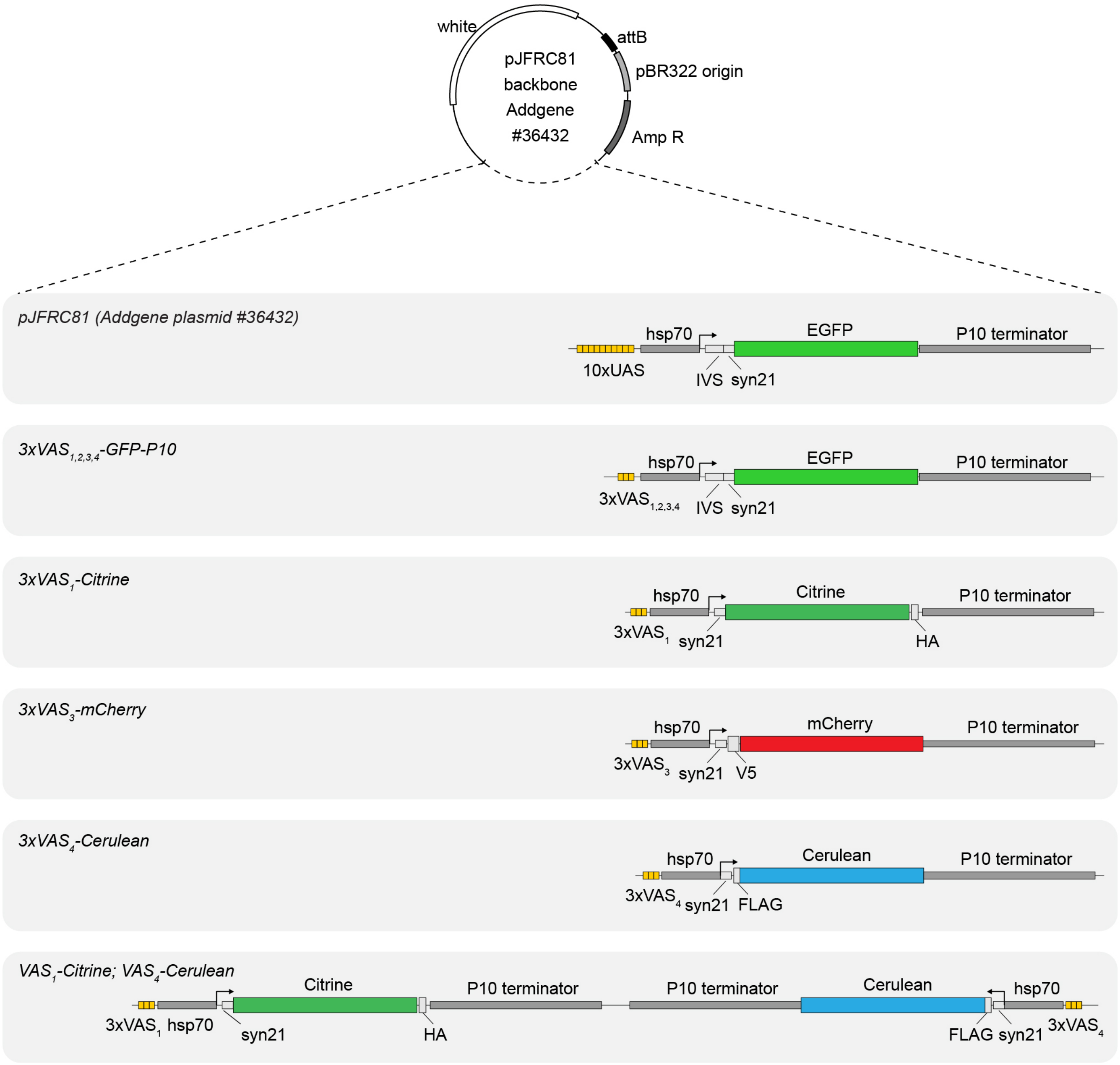

Supplementary Figure 2. VAS-constructs based on the pJFRC81 plasmid backbone. All VAS constructs used in this study were derived from the pJFRC81 plasmid (Addgene #36432). The cassette containing 10 x UAS copies was replaced with custom 3 x VAS copies matching the designed TALE drivers. In addition, the EGFP reporter gene was exchanged for Citrine, mCherry, or Cerulean. The Citrine / Cerulean cassettes were subsequently combined into a single vector (see Methods).

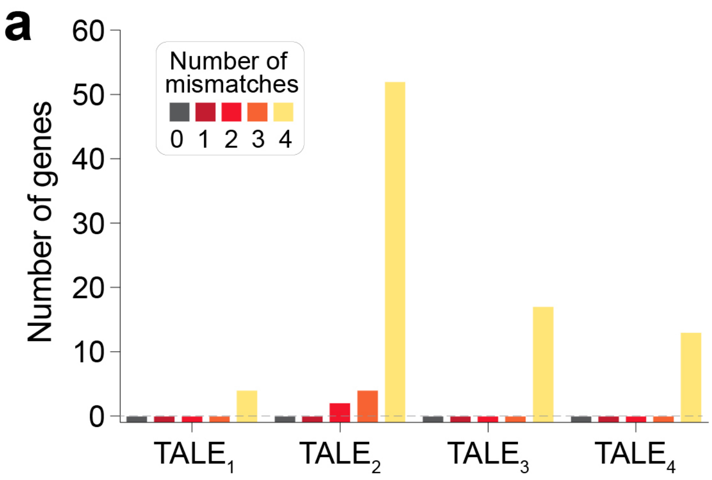

b

No. of mismatches

	0	1	2	3	4
$TALE_1$	0	0	0	0	4
$TALE_2$	0	0	2	4	52
$TALE_3$	0	0	0	0	17
$TALE_4$	0	0	0	0	13

Supplementary Figure 3. TALE off-target analysis. (a, b) Genome-wide *in silico* prediction of off-target sites containing up to four mismatches within a 4kb window (-2kb to + 2kb) centred on the transcription start sites (TSS) of annotated *Drosophila* genes. Bar graph (a) and matrix (b) show the number of putative target genes relative to the number of mismatches for each of the four TALEs.

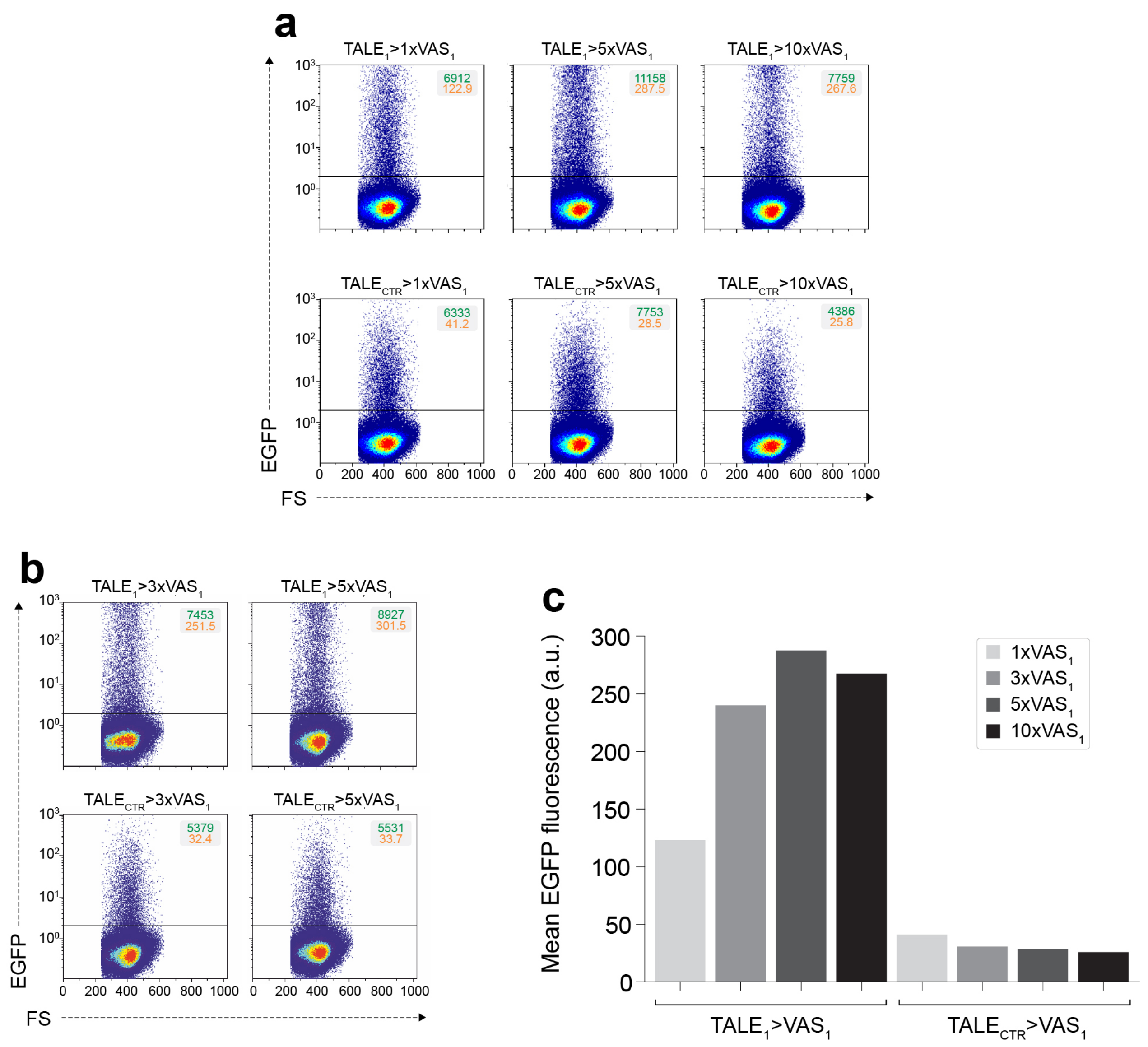

Supplementary Figure 4. Impact of VAS repeat number on reporter expression. (a) Flow cytometry scatter plots of reporter gene expression (EGFP channel) in pMT-GAL4 S2 cells co-transfected with $TALE_1$ driver and VAS_1 responder constructs containing either 1, 5, or 10 VAS repeats (top row). Background activation of the same responders was quantified with a TALE control construct ($TALE_{CTR}$, bottom row). **(b)** Same experimental conditions as in (a) using VAS1 responders containing 3 or 5 VAS repeats. **(c)** Bar graph compiled from the two independent experiments in (a) and (b). The levels of expression were normalized to the 5xVAS condition, which was tested in both experiments (a.u. = arbitrary units). Each bar represents a single transfection. Activation of EGFP reporter expression starts to saturate between 3x and 5xVAS repeats. Flow cytometry plot insets display number of cells within the EGFP+ve gate (green) and mean EGFP fluorescence intensity for this population (orange).

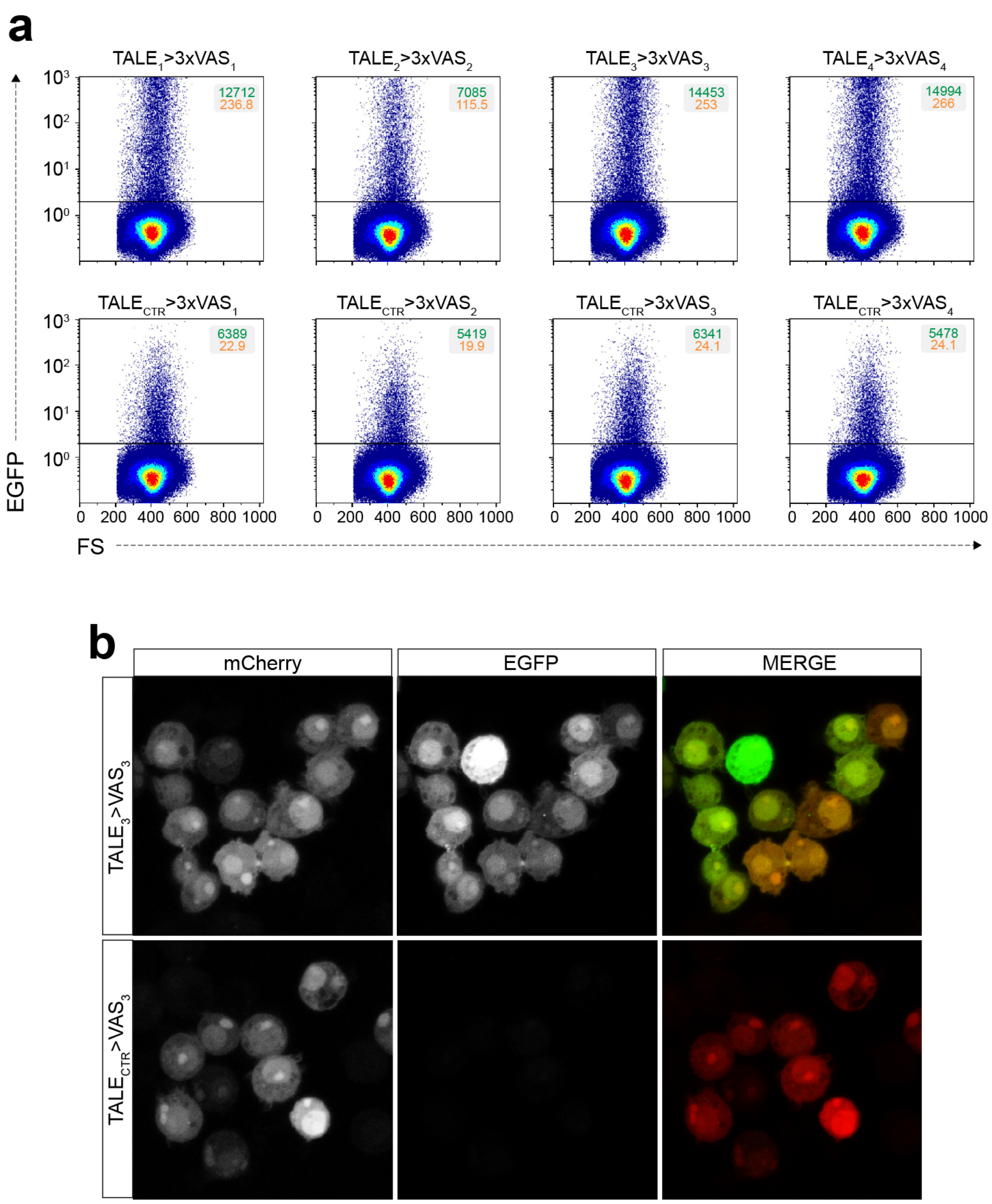

Supplementary Figure 5. Transgene induction by various TALE-VAS driver-reporter pairs. (a) Representative flow cytometry scatter plots of reporter expression (EGFP channel) driven by four different TALE-VAS pairs in pMT-GAL4 S2 cells (top row). Background expression levels were assessed using a TALE control construct ($TALE_{CTR}$, bottom row). TALEs 1, 3, and 4 exhibited strongest activation and were used in all subsequent experiments (FS = forward scatter). Flow cytometry plot insets display number of cells within the EGFP+ve gate (green) and mean EGFP fluorescence intensity for this population (orange). **(b)** Representative confocal images of pMT-GAL4 S2 cells transfected with either a cognate TALE-VAS pair ($TALE_3$>VAS_3) or the control $TALE_{CTR}$-VAS_3 pair, illustrating specific TALE-mediated activation of EGFP expression. mCherry was expressed from a separate plasmid and was used as transfection control. All images were recorded using the same settings with the intensity adjusted to non-saturating conditions.

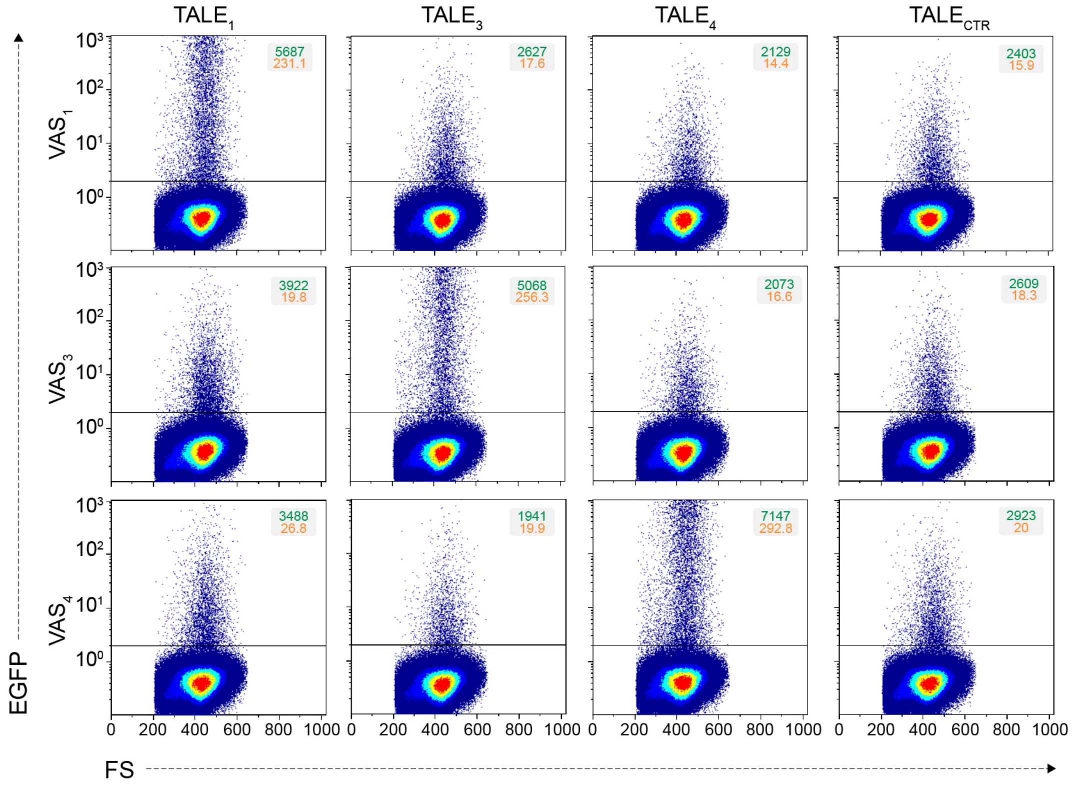

Supplementary Figure 6. Evaluation of TALE-VAS specificity in *Drosophila* cells. Representative flow cytometry scatter plots of reporter expression (EGFP channel) in pMT-GAL4 S2 cells transfected either with cognate TALE-VAS pairs, non-cognate TALE-VAS pairs, or $TALE_{CTR}$-VAS controls. EGFP activation above background levels ($TALE_{CTR}$-VAS conditions) was only observed with cognate TALE-VAS pairs (FS = forward scatter).Flow cytometry plot insets display number of cells within the EGFP+ve gate (green) and mean EGFP fluorescence intensity for this population (orange).

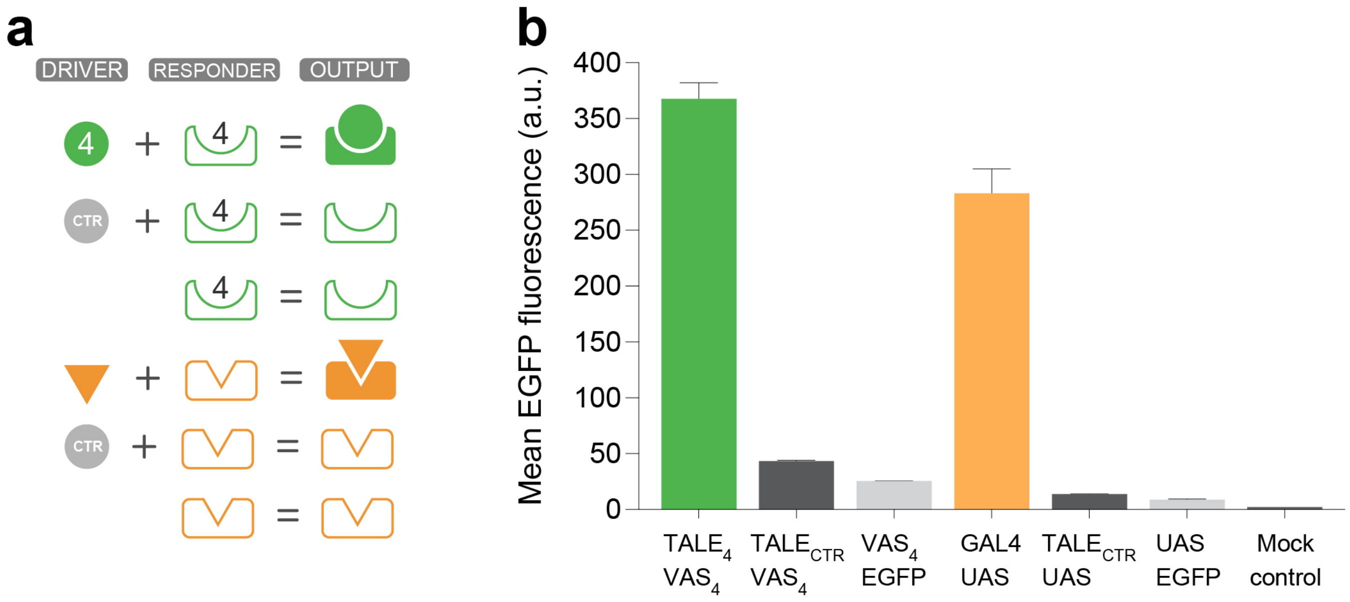

Supplementary Figure 7. Characterization of VAS and UAS responders in *Drosophila* S2 cells. (a) Schematic representation of the driver-responder combinations used. TALE driver constructs are depicted as circles (number 4 indicates $TALE_4$, CTR stands for $TALE_{CTR}$) and the GAL4 driver as a triangle. Corresponding responders have matching colours, numbers, and indentations. **(b)** Flow cytometry analysis of *Drosophila* S2 pMT-GAL4 cells transfected with various driver and responder combinations. Bar graph shows quantification of EGFP reporter expression in the presence of matching drivers ($TALE_4$ or GAL4), control driver ($TALE_{CTR}$), in the absence of any driver (responders alone), and mock transfected cells. In all cases, mean EGFP fluorescence was calculated from three biological replicates (n = 3 from one experiment, mean +/- s.d.; a.u., arbitrary units).

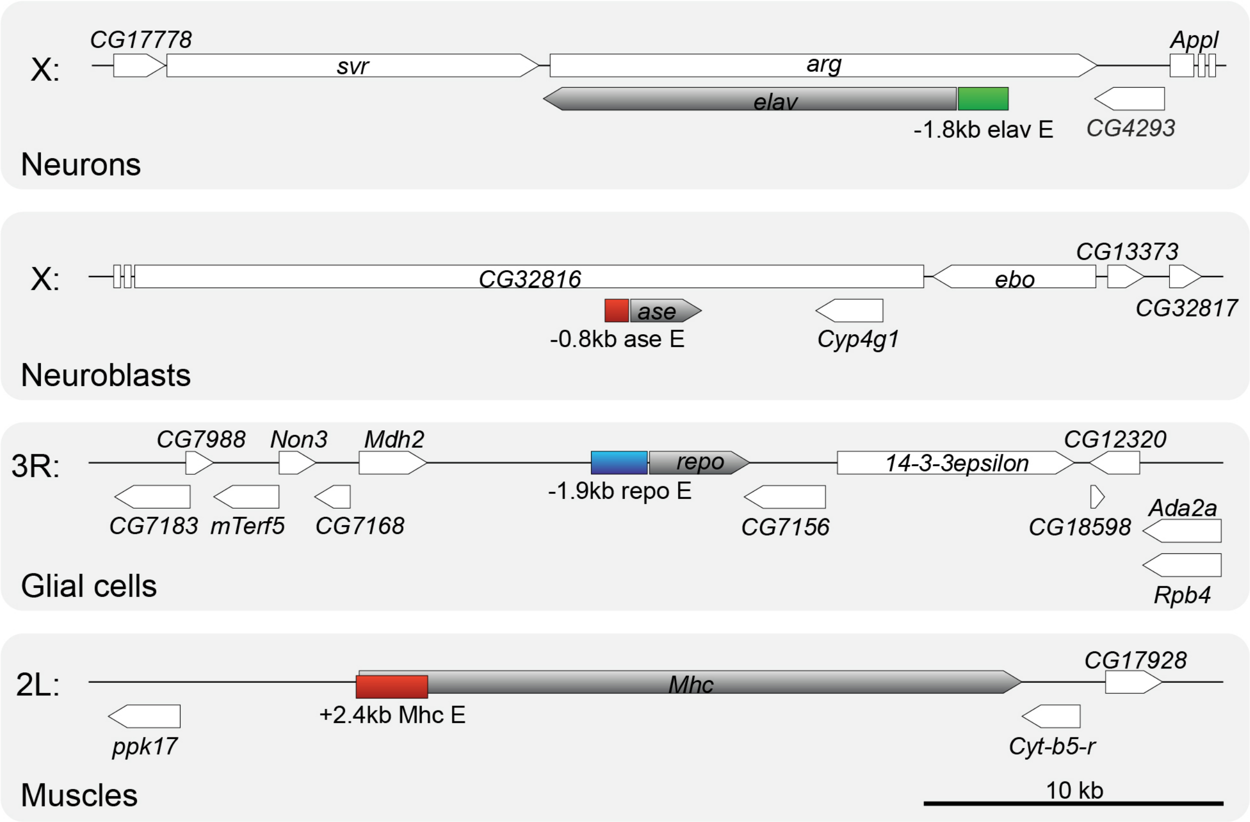

Supplementary Figure 8. Genomic maps and coordinates of enhancer sequences used to generate TALE drivers. Four enhancer elements were chosen to target transgene expression in larval neurons, neuroblasts, glial cells and muscles. The 1.8kb element upstream of the *elav* gene was used to generate the $elav^{1.8kb}$-$TALE_1$ driver for neuronal expression; the 0.8kb element upstream of the *asense* gene was used to generate the $ase^{0.8kb}$-$TALE_3$ driver for neuroblast expression; the 1.9kb element upstream of the *repo* gene was used to generate the $repo^{1.9kb}$-$TALE_4$ driver for glial cell expression; the 2.4kb element overlapping the start of the *Mhc* gene was used to generate the $mhc^{2.4kb}$-$TALE_3$ driver for muscle expression. The position of each enhancer element (E) in relation to the surrounding genes and their chromosomal location is shown. Diagrams were adapted from FlyBase:GBrowse.

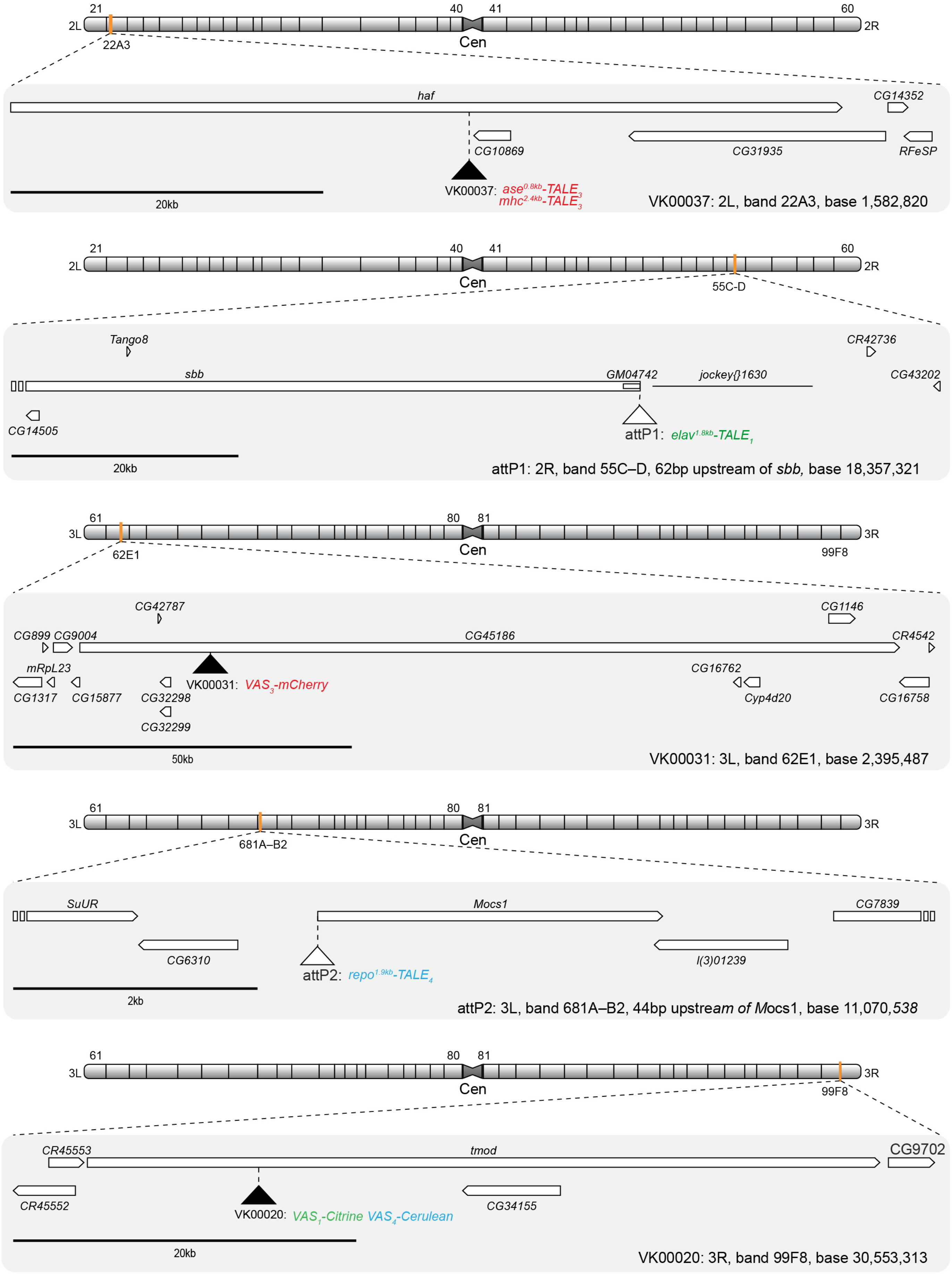

Supplementary Figure 9. Chromosomal insertions maps for the TALE drivers and VAS transgenes used in this study. Chromosomal maps showing the location of landing sites used for phiC31-mediated integration. Numbers above chromosomes indicate cytological bands. Black triangles reflect the position of piggyBac elements and white triangles of P-elements. VK00020, VK00031, and VK00037 are located in introns of coding genes while attP1 and attP2 map to intergenic regions. Cen = centromere. Diagrams were adapted from UCSC Genome Browser and FlyBase:GBrowse.

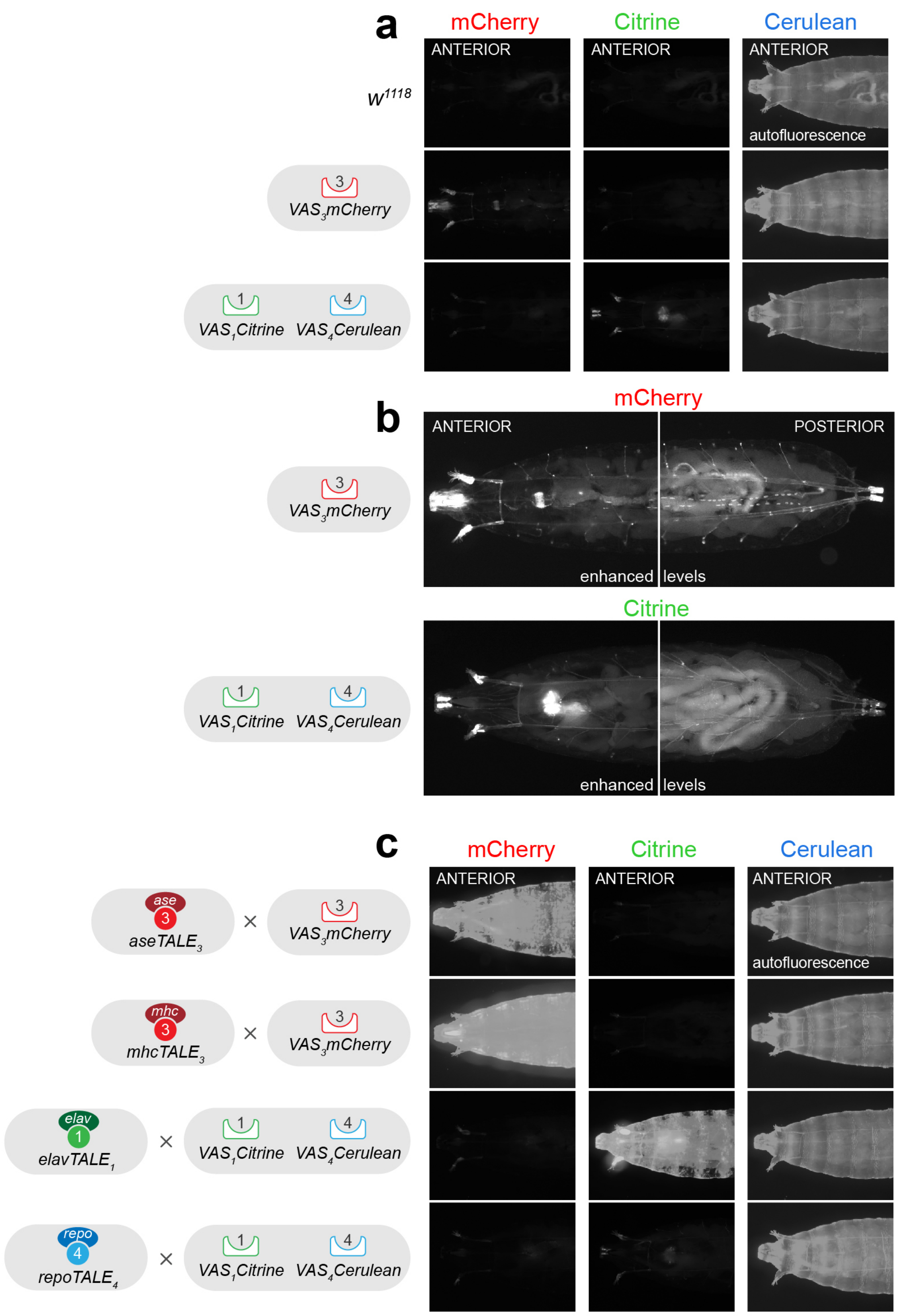

Supplementary Figure 10. Characterization of TALE driver and VAS responder lines. All panels show epifluorescence images of immobilized wandering third larval instars recorded at long exposure (7 seconds) to simulate the perception of the human eye. **(a)** Compared to the wild-type (w^{1118}) control, both *VAS*$_3$*-mCherry* and *VAS*$_1$*-Citrine* responders exhibit weak background expression in the mCherry and Citrine channels (see below). Background expression in the Cerulean channel could not be assessed due to strong autofluorescence of the larval cuticle at the corresponding excitation wavelength. **(b)** Images of the VAS responder lines in (a) were edited to enhance any background signal using the levels function in Adobe Photoshop. *VAS*$_3$*-mCherry* shows background expression in the feeding apparatus (mouth hooks), anterior and posterior spiracles, the trachea, the proventriculus, and the pericardial cells. *VAS*$_1$*-Citrine* and *VAS*$_4$*-Cerulean* exhibits background expression in the anterior part of the feeding apparatus, the anterior spiracles, the trachea, the central part of the brain and the ventral nerve cord (VNC). **(c)** The three drivers (*ase*$^{0.8kb}$*-TALE*$_3$, *mhc*$^{2.4kb}$*-TALE*$_3$*, and elav*$^{1.8kb}$*-TALE*$_1$) induce ectopic expression in the epidermis in addition to their predicted expression pattern.

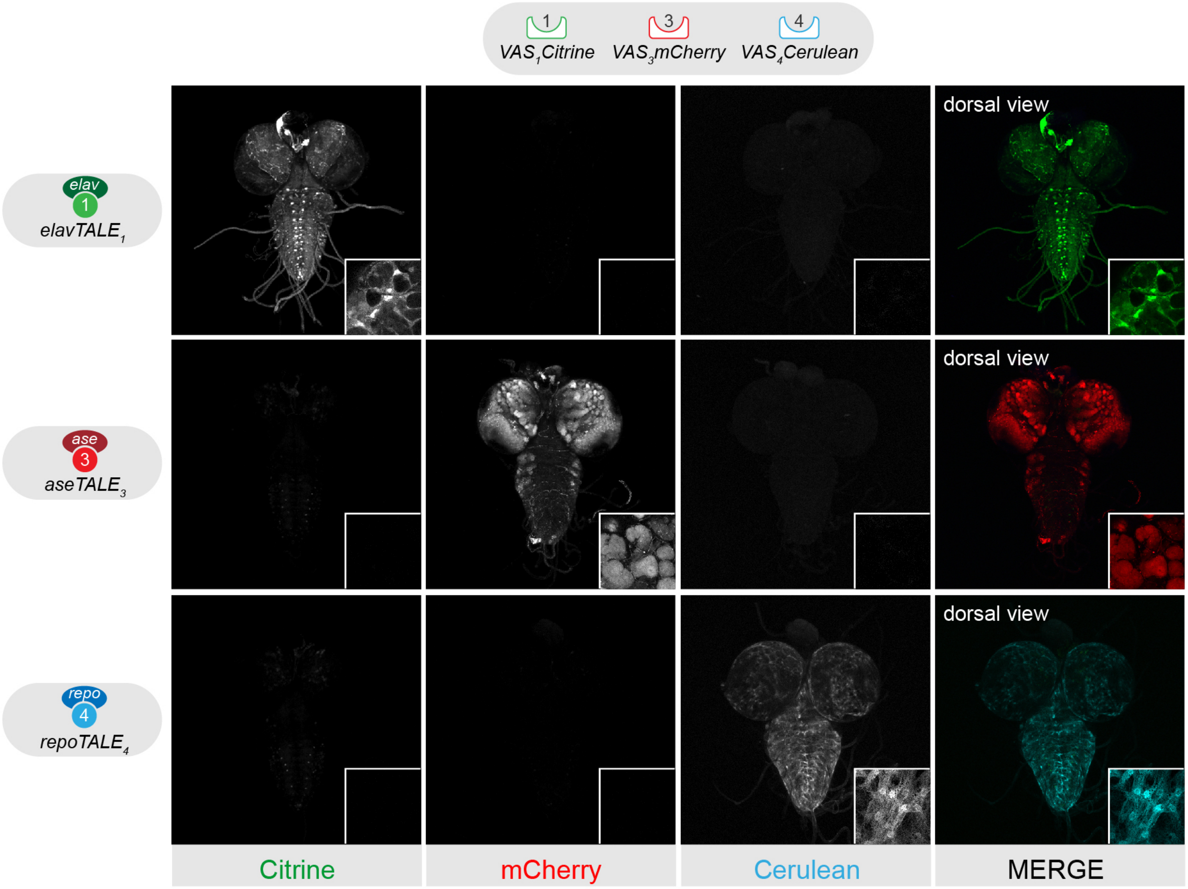

Supplementary Figure 11. Evaluation of cross-activation between TALE drivers and VAS responders in the *Drosophila* larval CNS. Each driver line, *elav$^{1.8kb}$-TALE$_1$*, *ase$^{0.8kb}$-TALE$_3$*, and *repo$^{1.9kb}$-TALE$_4$*, was crossed individually to the triple VAS responder line (*VAS$_1$-Citrine, VAS$_3$-mCherry, VAS$_4$-Cerulean*) and transgene expression was assessed by confocal microscopy in freshly dissected third larval instar brains. Insets show higher magnifications of neurons, neuroblasts and glial cells expressing Citrine, mCherry and Cerulean transgenes under the corresponding TALE-VAS pair combinations. All images were recorded using the same settings with the intensity adjusted to non-saturating conditions. TALE-VAS pairs drive transgene expression only in the target tissue and no background signal could be detected in neighbouring, interacting cells.

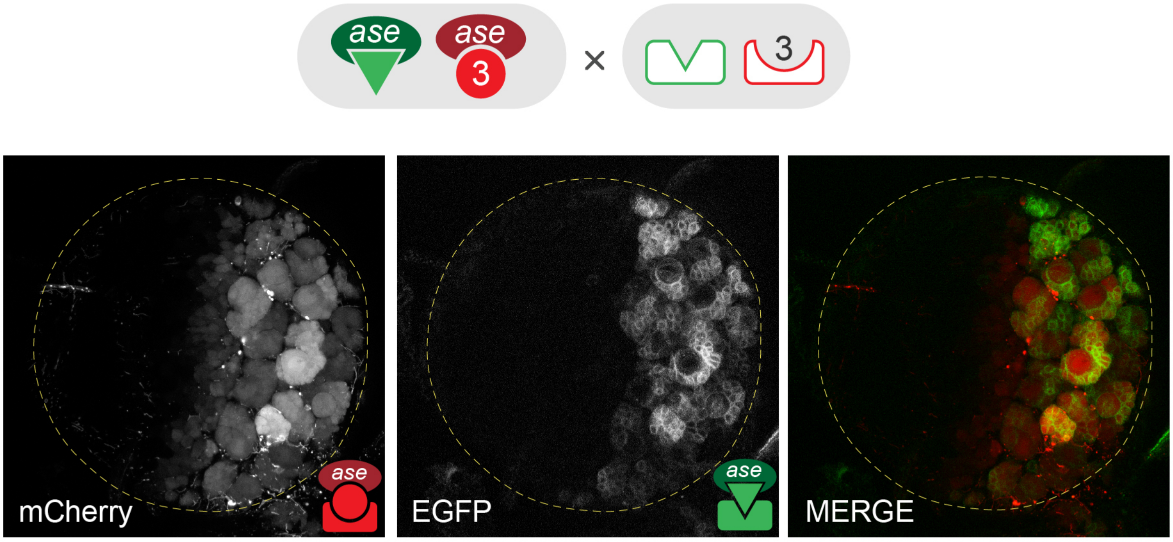

Supplementary Figure 12. Direct comparison of the TALE-VAS and GAL4-UAS systems in flies. High magnification confocal image of the ventral side of an optic lobe (dashed outline) from a third larval instar brain (*ase*$^{0.8kb}$-*TALE*$_3$; *VAS*$_3$-*V5-mCherry; UAS-mCD8-GFP; ase-GAL4*). Although both systems show varying signal intensities between individual neuroblast clusters, their overall expression pattern is comparable and overlaps in the majority of the cells.

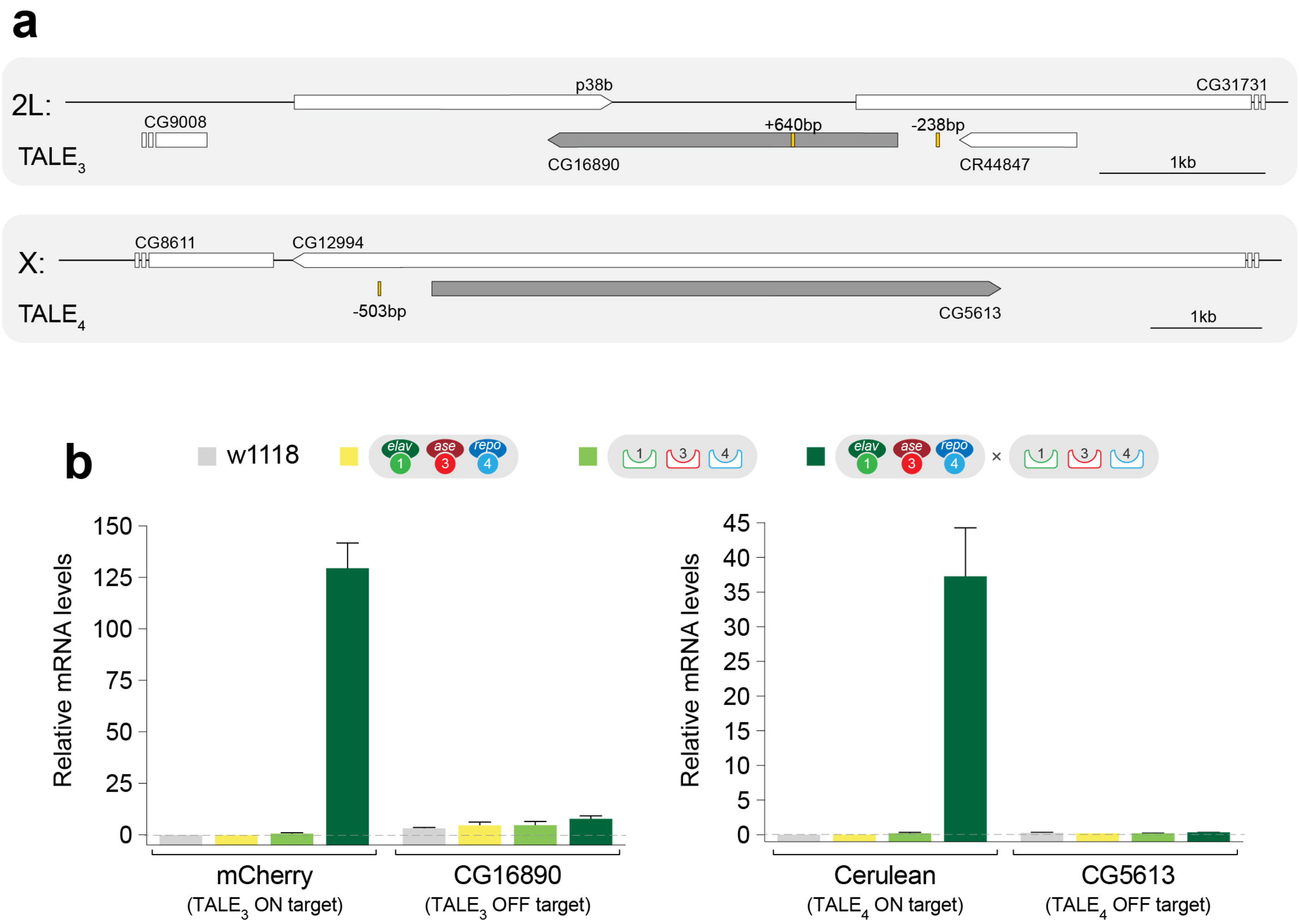

Supplementary Figure 13. Analysis of putative off-target sites *in vivo*. (a) Genomic locations of candidate off-target loci for $TALE_3$ (*CG16890*) and $TALE_4$ (*CG5613*) (diagrams adapted from FlyBase:GBrowse). The number of base pairs reflects the distance between the off-target site and the TSS of predicted target genes. All off-target loci contained four nucleotide mismatches relative to the corresponding on-target sequence. **(b)** Quantification of relative expression (RT-qPCR) from the on-target transgenes and off-target endogenous genes in wild-type (w^{1118}, grey), triple TALE driver (yellow), triple VAS responder (light green), and triple TALE driver>triple VAS responder (dark green) third larval instars (n = 3 biological replicates (x3 technical replicates); mean +/- s.d.).

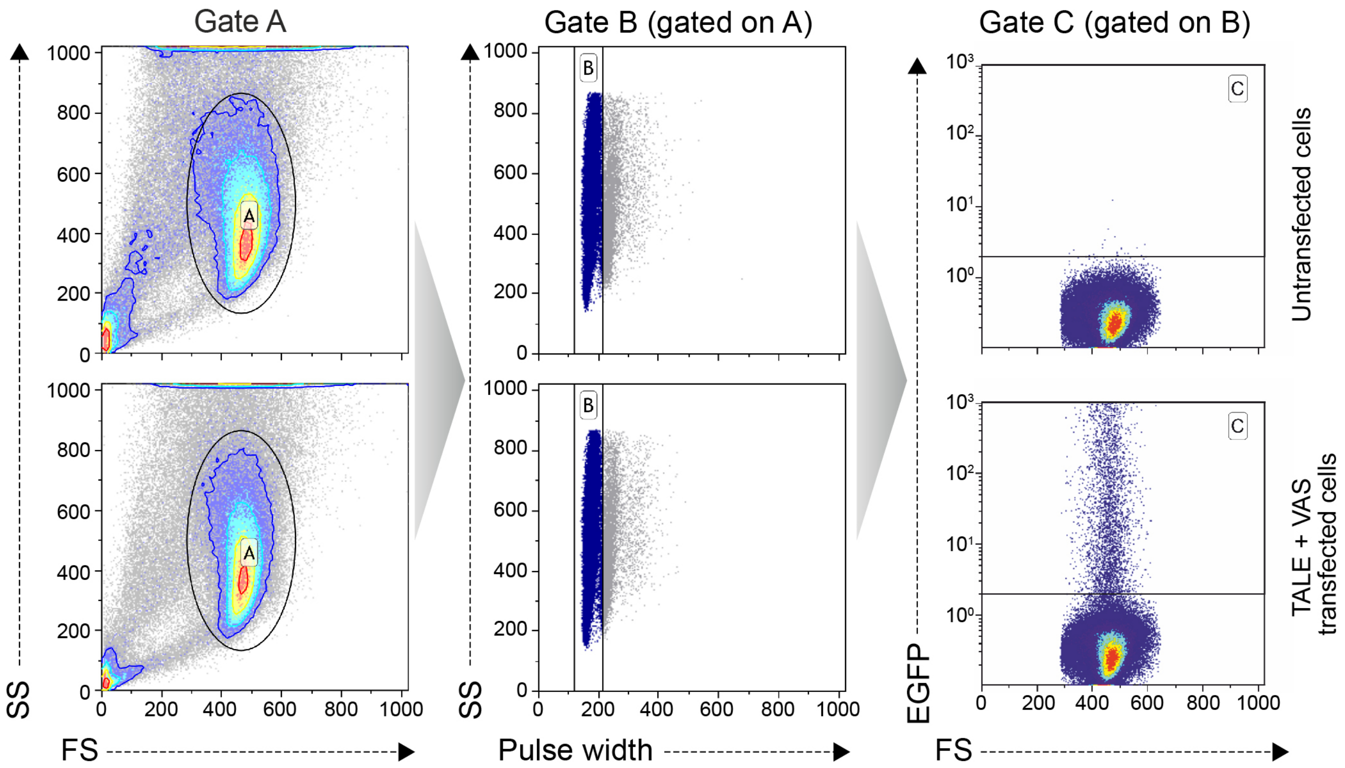

Supplementary Figure 14. Flow cytometry gating strategy. *Drosophila* S2 cells *were* first gated based on side scatter (SS) and forward scatter (FS) to select viable cells of approximately the right size (Gate A). A second gate was then set on Gate A to separate single cells from doublets and triplets (Gate B). Finally, a third gate (Gate C) was introduced using untransfected cells as a baseline to detect EGFP expressing cells within the population of cells in Gate B.
