# Supplementary material for: A multiplexable TALE-based binary expression system for in vivo cellular interaction studies
Source: Nat Commun. 2017 Nov 21;8:1663. doi: 10.1038/s41467-017-01592-3 (PMC5698491; doi:10.1038/s41467-017-01592-3)
Supplement: Supplementary file 3 — Description of Additional Supplementary Files [file 41467_2017_1592_MOESM3_ESM.pdf]

Description of Additional Supplementary Files

File Name: Supplementary Data 1
Description: **Predicted TALE driver off-target sites across the Drosophila genome.** Off-target sites containing up to 4 mismatches within a 4kb window (-2kb upstream to +2kb downstream) of the annotated transcription start sites (TSS) are listed together with the corresponding gene names, Ensemble ID, number of mismatches, and distance to the TSS. Binding orientation is indicated on the forward and reverse strands.

File Name: Supplementary Data 2
Description: **Variable upstream sequences (VAS) and corresponding TALEs.** All VAS contain a 5'-T preceding the TALE array (parantheses, green) and an obligatory T at the 3' end (red). TALE monomers are indexed according to their position in the respective starting vector (pFUS_A, pFUS_B8). The terminal NG is added during the second cloning step (Cermak et al., 2011).

File Name: Supplementary Data 3
Description: **List of DNA primers and oligos used in this study.** All oligonucleotides were synthesized by Integrated DNA Technologies (IDT).

File Name: Supplementary Data 4
Description: Genotypes of fly stocks generated in this paper.
